# Licochalcone A, a Polyphenol Present in Licorice, Suppresses UV-Induced COX-2 Expression by Targeting PI3K, MEK1, and B-Raf

**DOI:** 10.3390/ijms16034453

**Published:** 2015-02-20

**Authors:** Nu Ry Song, Jong-Eun Kim, Jun Seong Park, Jong Rhan Kim, Heerim Kang, Eunjung Lee, Young-Gyu Kang, Joe Eun Son, Sang Gwon Seo, Yong Seok Heo, Ki Won Lee

**Affiliations:** 1 WCU Biomodulation Major, Center for Food and Bioconvergence, Department of Agricultural Biotechnology, Seoul National University, Seoul, 151-742, Korea; E-Mails: happynury@gmail.com (N.R.S.); idonlike@gmail.com (J.-E.K.); allmendhubel@gmail.com (J.R.K.); heeerim@gmail.com (H.K.); thief28@snu.ac.kr (E.L.); joe8601@gmail.com (J.E.S.); seo0414@naver.com (S.G.S.); 2 Advanced Institute of Convergence Technology, Seoul National University, Suwon, 443-270, Korea; 3 Skin Research Institute, Amorepacific R&D Center, Yongin, 446-829, Korea; E-Mails: superbody@amorepacific.com(J.S.P.); kangyg82@amorepacific.com (Y.-G.K.); 4 Traditional Alcoholic Beverage Research Team, Korea Food Research Institute, Seongnam 463-746, Korea; 5 Department of Chemistry, Konkuk University, Seoul, 143-701, Korea; E-Mail: ysheo@konkuk.ac.kr

**Keywords:** Licochalcone A, Solar ultraviolet, Cyclooxygenase-2, PI3K, MEK1, B-Raf

## Abstract

Licorice is a traditional botanical medicine, and has historically been commonly prescribed in Asia to treat various diseases. Glycyrrhizin (Gc), a triterpene compound, is the most abundant phytochemical constituent of licorice. However, high intake or long-term consumption of Gc has been associated with a number of side effects, including hypertension. However, the presence of alternative bioactive compounds in licorice with anti-carcinogenic effects has long been suspected. Licochalcone A (LicoA) is a prominent member of the chalcone family and can be isolated from licorice root. To date, there have been no reported studies on the suppressive effect of LicoA against solar ultraviolet (sUV)-induced cyclooxygenase (COX)-2 expression and the potential molecular mechanisms involved. Here, we show that LicoA, a major chalcone compound of licorice, effectively inhibits sUV-induced COX-2 expression and prostaglandin E2 PGE_2_ generation through the inhibition of activator protein 1 AP-1 transcriptional activity, with an effect that is notably more potent than Gc. Western blotting analysis shows that LicoA suppresses sUV-induced phosphorylation of Akt/ mammalian target of rapamycin (mTOR) and extracellular signal-regulated kinases (ERK)1/2/p90 ribosomal protein S6 kinase (RSK) in HaCaT cells. Moreover, LicoA directly suppresses the activity of phosphoinositide 3-kinase (PI3K), mitogen-activated protein kinase kinase (MEK)1, and B-Raf, but not Raf-1 in cell-free assays, indicating that PI3K, MEK1, and B-Raf are direct molecular targets of LicoA. We also found that LicoA binds to PI3K and B-Raf in an ATP-competitive manner, although LicoA does not appear to compete with ATP for binding with MEK1. Collectively, these results provide insight into the biological action of LicoA, which may have potential for development as a skin cancer chemopreventive agent.

## 1. Introduction

**Figure 1:**
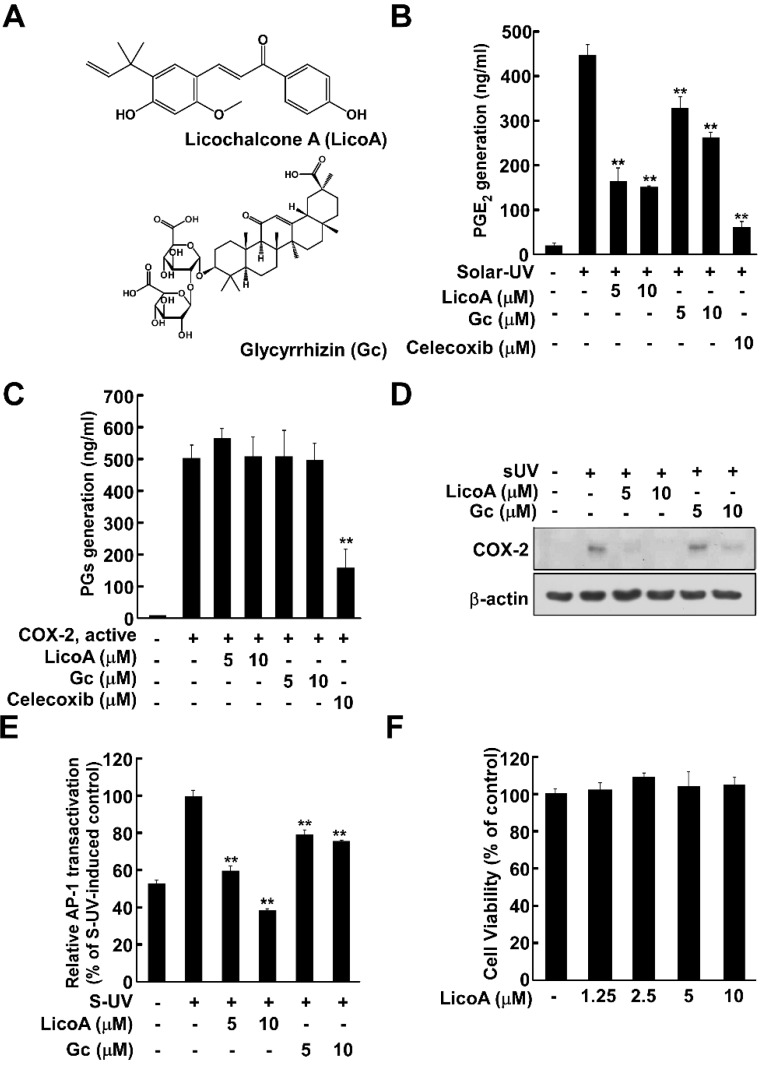
Treatment with licochalcone A (LicoA) but not Glycyrrhizin (Gc) inhibits solar ultraviolet (sUV)-induced prostaglandin E2 PGE_2_ production by suppressing cyclooxygenase (COX)-2 expression in HaCaT cells. (**A**) Chemical structure of LicoA and Gc; (**B**) LicoA suppressed sUV-induced PGE_2_ generation. HaCaT cells were treated with LicoA, Gc, and celecoxib at the indicated concentrations for 1 h before exposure to SUV, and harvested 24 h later. PGE_2_ production was measured using a PGE_2_ assay kit as described in the Experimental Section; (**C**) Treatment with LicoA or Gc did not suppress COX-2 activity. COX-2 activity was measured as described in the Experimental Section; (**D**) LicoA suppressed sUV-induced COX-2 expression in HaCaT cells. Cells were more potently inhibited by LicoA than Gc. Data is representative of 3 independent experiments that yielded similar results; (**E**) LicoA suppressed sUV-induced AP-1 transactivation in HaCaT cells. For the luciferase assay, HaCaT cells stably transfected with AP-1-luciferase reporter plasmids were cultured as described in the Materials and Methods. The cells were then starved in 0.1% fetal bovine serum (FBS)/minimal essential medium (MEM) in the presence or absence of LicoA or Gc at the indicated concentrations (5, 10 μM) for 1 h before exposure to sUV for 6 h. Luciferase activity was then assayed. activator protein 1 AP-1 activity is expressed relative to that of the control cells (without sUV irradiation). Data are presented as mean AP-1 luciferase activity ± SD calculated from three independent experiments; (**F**) The effect of LicoA was not attributable to any detectable effects on HaCaT cell viability. Cells were treated with 1.25, 2.5, 5, or 10 μM LicoA for 1 h before sUV radiation for 24 h. Cell viability was measured using (3-(4,5-dimethylthiazol-2-yl)-5-(3-carboxymethoxyphenyl)-2-(4-sulfophenyl)-2H-tetrazolium) (MTS) assay. Data are shown as mean ± S.D. and asterisks indicate significant inhibition by LicoA or Gc compared to the group treated with sUV alone (** *p* < 0.01).

Licorice root is a traditional herbal medicine and one of the most commonly prescribed botanicals in East Asia for the traditional treatment of various diseases including inflammation, gastric ulcers, atherosclerosis and cancer [1,2,3]. Licorice root and licorice extract contain essential oils, alkaloids, polysaccharides, polyamines, triterpenes, and flavonoids [4]. Glycyrrhizin (Gc, Figure 1A) is a triterpene compound, and the most abundant constituent of licorice, comprising between 3.63% and 13.06% of dry root content [3]. However, high intake or long-term consumption of Gc has been associated with several deleterious effects including hypertension, hypertensive encephalopathy, hypokalemia, and suppression of the rennin aldosterone system [5,6,7]. Therefore, other bioactive compounds may be more appropriate for pharmaceutical or neutraceutical development. Licochalcone A (LicoA, Figure 1A) is a major chalcone present in licorice root and has anti-parasitic, antibacterial and anti-tumor properties [8]. However, to date, there have been no reports on the chemopreventive effect of LicoA against solar ultraviolet (sUV)-induced cyclooxygenase (COX)-2 expression or the potential molecular mechanisms involved.

COX-2 is an essential enzyme that mediates the conversion of arachidonic acid to prostaglandin, the inducible isoform of cyclooxygenase [9]. The inflammatory process is known to influence human malignancies, including skin cancer, by promoting epidermal hyperproliferation and hyperplasia through the release of various inflammatory factors including prostaglandin E2. Data from mouse models has shown that COX-2 over-expression occurs in hyperplastic skin, benign tumors, and malignant tumors following chronic UVB irradiation [10]. UV light is a well-established carcinogen that produces squamous-type tumors in mouse skin [11]. UV light acts as both an initiator (presumably by causing DNA damage leading to gene mutations) and as a tumor promoter [12]. Because UV irradiation cannot penetrate further than the skin in humans, this organ is the primary site of UV light-induced damage and carcinogenesis. sUV light can be very harmful to human health, causing DNA damage, inflammation, sunburn, immunosuppression, photoaging, gene mutations, and skin cancer [13]. Therefore, the regulation of COX-2 expression could represent a promising strategy for protection against skin cancer.

The idea of targeting multiple signaling pathways has emerged as a prominent approach for innovative and effective therapeutic strategies for skin cancer. Major signaling pathways that are known to mediate UV-induced biological responses include mitogen-activated protein kinases (MAPKs) and phosphatidylinositol-3 kinase (PI3K) [11,14]. The MAPK and PI3K/Akt signaling pathways play important roles in many biological processes, including inflammation, apoptosis, proliferation, and differentiation. These kinases are activated by UV exposure via specific upstream kinases including Raf and MAPK/ extracellular signal-regulated kinases (ERK) kinase (MEK) 1/2, which in turn activate ERKs, PI3K and Akt [15]. Because MAPKs and PI3K/Akt are the primary mediators of UV-induced COX-2 expression [11], the inhibition of enzymes in these signaling pathways may reduce COX-2 expression, representing a potentially powerful strategy for preventing the harmful effects of UV irradiation.

In the present study, we investigated the chemopreventive effects of LicoA on sUV-induced tumor promotion and examined the underlying molecular mechanisms involved. Here, we report that LicoA suppresses sUV-induced COX-2 expression by acting as a potent direct physical inhibitor of PI3K, MEK1, and B-Raf.

## 2. Results

### 2.1. Licochalcone A (LicoA) Inhibits Cyclooxygenase (COX)-2 Expression and Suppresses Solar Ultraviolet (sUV)-Induced prostaglandin E2 PGE_2_ Generation More Potently than Glycyrrhizin (Gc)

COX-2 is widely reported to be one of the most important inflammatory mediators associated with UV-induced skin cancer [10], so we first examined the effect of LicoA on sUV-induced PGE_2_ generation, a primary outcome of COX-2 expression. In HaCaT cells, sUV exposure induced PGE_2_ generation, which was suppressed by LicoA treatment, and to an effect greater than that of Gc (Figure 1B). Celecoxib, a well-known commercial COX-2 inhibitor, was used as a positive control. Because LicoA and Gc both reduced PGE_2_ generation, we next sought to determine their effects on COX-2 enzyme activity. Neither compound had any observable effect in this regard (Figure 1C). We next examined the effects of LicoA on sUV induced COX-2 expression. LicoA suppressed sUV-induced COX-2 expression levels to an extent greater than that of Gc (Figure 1D). Because AP-1 is a major transcriptional regulator of COX-2 expression, we analyzed the effect of LicoA treatment on sUV-induced transactivation of AP-1 using HaCaT cells stably transfected with AP-1 promoter–luciferase reporter plasmids. LicoA was observed to inhibit the sUV-induced transactivation of AP-1 in a dose-dependent manner. Although Gc also suppressed sUV-induced COX-2 expression, its effect was weaker than that of LicoA. It remains plausible that the ability of LicoA to inhibit AP-1 transactivation may underlie its anti-inflammatory and anti-carcinogenic effects (Figure 1E). Treatment of LicoA did not induce cell cytotoxicity up to concentrations of 10 μM, indicating that the inhibitory effect of LicoA on sUV-induced COX-2 expression was not due to cytotoxicity (Figure 1F).

### 2.2. Lico A Inhibits sUV-Induced Phosphorylation of Akt/ mammalian target of rapamycin (mTOR) and mitogen-activated protein kinase kinase (MEK)1/ extracellular signal-regulated kinases (ERKs)/p90 ribosomal protein S6 kinase (RSK) Pathways in HaCaT Cells

**Figure 2:**
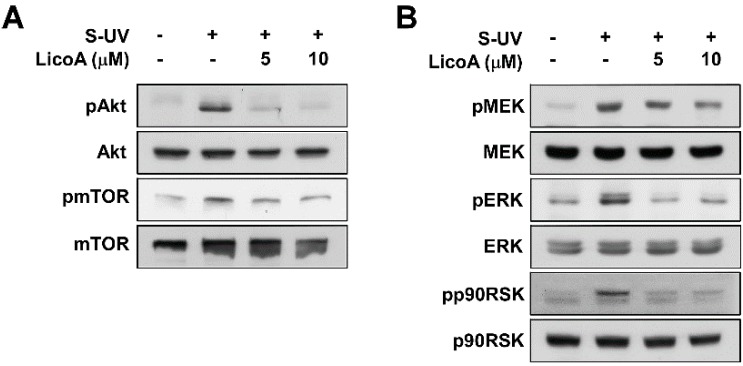

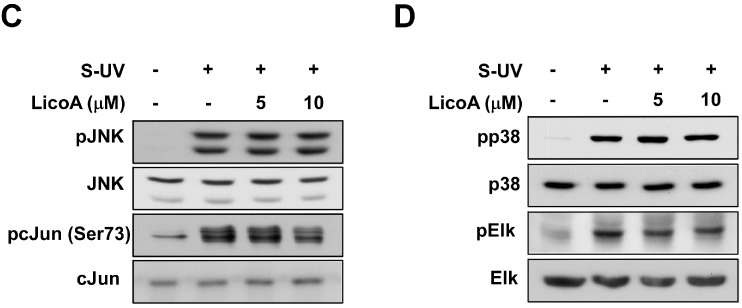
Effects of LicoA on sUV-dependent phosphorylation of the Akt/ mammalian target of rapamycin (mTOR), mitogen-activated protein kinase kinase (MEK)1/ extracellular signal-regulated kinases (ERKs)/p90 ribosomal protein S6 kinase (RSK), c-Jun *N*-terminal kinases (JNK)/c-Jun, and p38/Elk pathways in HaCaT cells. Cells were treated with 1.25, 2.5, 5, or 10 μM LicoA for 1 h before sUV radiation, and harvested 30 min later. Phosphorylation levels as well as total mitogen-activated protein kinases (MAPKs) and Akt protein content were determined by Western blot analysis, as described in the Materials and Methods, using antibodies specific for the corresponding phosphorylated and total proteins.

AP-1 is regulated by various signaling cascades, including the MAPK pathway. MAPKs phosphorylate and thereby activate activator protein 1 AP-1 subunits such as c-Jun. When we examined LicoA for its effects on sUV-induced MAPK signal transduction, we found that it suppressed both the UVB-induced phosphorylation of Akt/ mammalian target of rapamycin mTOR (Figure 2A) and ERKs/p90 ribosomal protein S6 kinase (RSK) (Figure 2B). We next examined the effect of LicoA on MEK1 phosphorylation, an upstream kinase of ERK1/2. LicoA did not suppress MEK1 phosphorylation, while sUV-induced phosphorylation of JNK/c-Jun and p38/Elk was also not inhibited by the treatment of LicoA (Figure 2C,D). Taken together, these results indicate that PI3Kand Raf kinases (an upstream regulator of Akt or MEK) may be potential molecular targets of LicoA.

### 2.3. LicoA Inhibits phosphoinositide 3-kinase PI3K, MEK1, and B-Raf Kinase Activity

**Figure 3:**
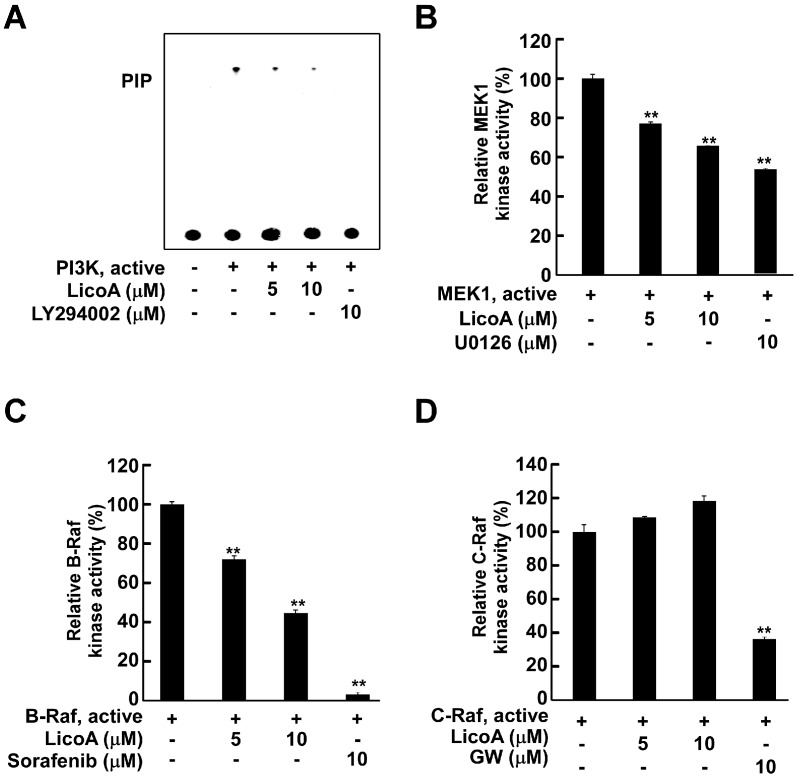
Effect of LicoA on phosphoinositide 3-kinase PI3K, MEK1, B-Raf, and C-Raf kinase activity. (**A**) LicoA inhibits PI3K activity. Active PI3K (100 ng) was preincubated with LicoA or LY294002 at the indicated concentrations for 10 min at 30 °C, then incubated with phosphatidylinositol substrate and [γ-^32^P]ATP for an additional 30 min at 30 °C. The resulting ^32^P-labeled phosphatidylinositol-3-phosphate (PIP) was measured as described in the Experimental Section; (**B**–**D**) LicoA inhibits MEK1 (**B**), and B-Raf (**C**), but not C-Raf (**D**) kinase activity *in vitro*. The MEK1, B-Raf, and C-Raf *in vitro* kinase assays were performed as described in the Experimental Section, and kinase activity is expressed as percent inhibition relative to the activity of the untreated kinase control. The average ^32^P count was determined from three separate experiments, and the data are presented as the mean values ± S.D. ** *p* < 0.01.

To confirm whether PI3K, MEK1, and Raf are plausible molecular targets of LicoA in the inhibition of cell proliferation, we next determined their effects on these proteins and C-Raf kinase activity *in vitro*. Treatment of LicoA significantly suppressed PI3K (Figure 3A) in a dose-dependent manner. LY294002, a widely-used commercial PI3K inhibitor, was employed as a positive control. We also found that LicoA suppressed MEK1 (Figure 3B), and strongly blocked B-Raf (Figure 3C) activity in a dose-dependent manner. However, the activity of C-Raf, another isoform of Raf, did not show any change in the presence of LicoA (Figure 3D). These results suggest that PI3K, MEK1, and B-Raf are molecular targets of LicoA in the suppression of sUV-induced COX-2 expression in HaCaT cells.

### 2.4. LicoA Directly Binds to PI3K and B-Raf in an ATP-Competitive Manner and Interacts Directly with MEK1 in an ATP Non-Competitive Manner

**Figure 4:**
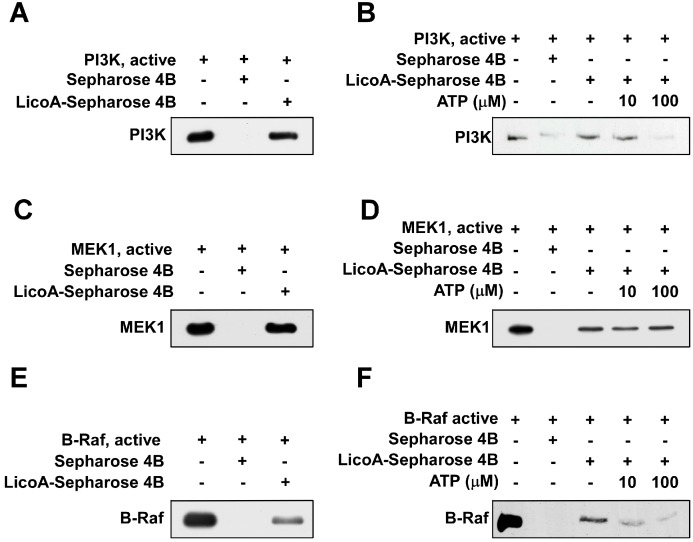
LicoA directly binds with PI3K, MEK1, and B-Raf. (**A**, **C** and **E**) LicoA specifically binds with PI3K (**A**), MEK1 (**C**), and B-Raf (**E**) *in vitro*. The PI3K (or MEK1, B-Raf)—LicoA binding was confirmed by immunoblotting using antibodies against PI3K(p110), MEK1 or B-Raf: Lane 1 (input control), PI3K, MEK1, or B-Raf protein standard; Lane 2 (control), Sepharose 4B was used to immunoprecipitate PI3K, MEK1, or B-Raf, as described in the Experimental Section; Lane 3, LicoA-Sepharose 4B affinity beads were used to immunoprecipitate PI3K, MEK1, or B-Raf. (**B**, **D** and **F**) LicoA competes with ATP to bind with PI3K and B-Raf but not MEK1. Active PI3K, MEK1, or B-Raf (200 ng) was incubated with ATP at different concentrations (0, 10, or 100 µM) and 100 µL of LicoA-Sepharose 4B or 100 µL of Sepharose 4B (negative control) in reaction buffer for a final volume of 500 µL. The mixtures were incubated at 4 °C overnight with shaking. After washing, the immunoprecipitated proteins were detected by Western blotting: Lane 1 (input control), PI3K, MEK1, or B-Raf protein standard; Lane 2 (negative control), PI3K, MEK1, or B-Raf bound with Sepharose 4B; Lane 3 (positive control), PI3K, MEK1, or B-Raf binding with LicoA—Sepharose 4B. Each experiment was performed a minimum of three times and representative blots are shown.

To determine whether LicoA exerts its effects by direct physical interaction with PI3K, MEK1, and B-Raf, we performed an immunoprecipitation assay using LicoA-conjugated Sepharose 4B beads. After immunoprecipitation, we detected PI3K (Figure 4A), MEK1 (Figure 4C), and B-Raf (Figure 4E) in reactions containing LicoA-Sepharose 4B conjugated beads, but not in reactions with Sepharose 4B beads alone. ATP treatment blocked the binding ability of LicoA with PI3K (Figure 4B) and B-Raf (Figure 4F) in a dose-dependent manner, suggesting that LicoA binds with PI3K and B-Raf in competition with ATP. However, the binding ability of MEK1 was not suppressed in the presence of ATP, indicating that LicoA binds with MEK1 in an ATP non-competitive manner (Figure 4B).

## 3. Discussion

Licorice is frequently prescribed as a herbal remedy in East Asia, and licorice root extract has been recognized by the National Cancer Institute as harboring chemopreventive properties [16]. Licorice root and licorice extract contain essential oils, alkaloids, polysaccharides, polyamines, triterpenes, and flavonoids [4]. Because Gc is quantitatively the most significant component of dried roots [3], it is normally considered to be the principal biologically active component of licorice. The most widely observed pharmacological properties of licorice triterpenoids, such as Gc, are hydrocortisone-like anti-inflammatory effects [17,18,19], and antioxidant activity [20]. Licorice root extract and Gc have been reported to inhibit tumorigenesis in skin, liver, lung, colon, and breast cancers [21,22,23,24,25]. A number of previous studies have demonstrated that Gc and glycyrrhetic acid (GA), a Gc aglycone, exhibit anti-tumor effects in a two-stage skin tumorigenesis animal model induced by 7,12-DMBA (dimethylbenz[*a*]anthracene) and 12-O-Tetradecanoylphorbol-13-acetate (TPA) [26,27,28,29]. However, both long term and high intake of Gc consumption have been associated with undesirable mineralocorticoid excess, hypertension, and hypokalemia [6,30].

In addition to triterpenoids, approximately 300 polyphenols in concentrations of 1%–5% in dried root have been isolated from *Glycyrrhiza* species. These include phenolic acids, flavones, flavans, chalcones, and isoflavonoids [31,32,33]. LicoA is a major chalcone compound present in the root of licorice and has anti-parasitic, antibacterial and anti-tumor properties [8]. Previous studies have demonstrated that LicoA has anti-tumorigenic effects through its ability to induce apoptosis and inhibit cell proliferation in gastric and prostate cancer cells [34,35,36]. Studies have also shown that LicoA has inhibitory effects on inflammatory processes by suppressing LPS signaling pathway *in vitro* and *in vivo* [37,38]. However, to date, there have been no reports on the suppressive effects of LicoA against sUV-induced COX-2 expression and its molecular targets in skin cancer cells. In the present study, we observed that LicoA had a more potent inhibitory effect than Gc on sUV-induced COX-2 expression in HaCaT cells.

The aberrant expression of COX-2 is frequently detected in epithelial cancers, including skin cancer in mice and humans [39], playing a key role in skin carcinogenesis. The inflammatory process affects human malignancies, including skin cancer, by promoting epidermal hyperproliferation and hyperplasia through the release of various inflammatory factors, such as prostaglandin E2. Previous studies have demonstrated that tumor incidence and aggressiveness induced by DMBA and TPA treatment are reduced in mice deficient for COX-2 [40,41]. Therefore, the inhibition of COX-2 over-expression represents a promising strategy for chemoprevention. We observed that LicoA suppresses sUV-induced COX-2 expression and PGE_2_ generation in HaCaT human keratinocytes. Although Gc also inhibited sUV-induced COX-2 expression and PGE_2_ generation, its effect was weaker than that of LicoA. UVB irradiation stimulates activator protein-1 (AP-1) a crucial transcription factor involved in COX-2 expression and linked to carcinogenesis [42,43], especially skin cancer development [44]. We found that LicoA did not suppress COX-2 enzyme activity *in vitro*, but inhibited AP-1 luciferase activity in HaCaT cells. These results show that LicoA suppresses sUV-induced COX-2 expression and PGE2 generation via transcriptional-level regulation, and that LicoA exerts potent anti-carcinogenic and anti-inflammatory effects.

The PI3K/Akt and MAPK pathways relay signals from the cell surface to activate transcription factors, thereby contributing to the regulation of target gene expression. Because sUV radiation activates PI3K/Akt, ERK1/2, p38, and JNK1/2, the successful inhibition of at least one of these MAPKs may partially attenuate the effects of UV irradiation. The PI3K/Akt and MAPK pathways are major signaling cascades that mediate UV-induced biological responses [11,14]. Inhibition of Akt phosphorylation by a PI3K inhibitor or dominant-negative Akt mutant has been shown to suppress UVB-induced COX-2 transcription in human keratinocytes [45]. It has been previously shown that MAPK pathways also play a crucial role in regulating UVB-induced COX-2 transcription in human keratinocytes [46,47]. In the present study, we found that LicoA effectively suppressed sUV-induced phosphorylation of Akt and mTOR, a downstream of effector of Akt sUV-induced phosphorylation of ERKs/p90RSK was also inhibited by LicoA. However, LicoA did not appear to influence the JNK1/2 or p38 signaling pathways. To elucidate the molecular mechanisms underlying these effects, we analyzed the phosphorylation status of upstream regulators of ERKs. LicoA marginally inhibited the sUV-induced phosphorylation of MEK1.

The most important limiting factor in target-based therapy is the narrow target specificity of the agents used, which may be overcome by targeting alternative kinase pathways that are also hyperactivated during cancer progression. The use of multitarget chemopreventive agents may be a useful tool for circumventing these limitations [48,49]. Two such multi-targeted kinase inhibitors (sunitinib and sorafenib) have proven effective in clinical testing [50]. The idea of targeting multiple signaling pathways has also emerged as a promising approach for the innovative and effective treatment of skin cancer. Because LicoA inhibits the Akt/mTOR signaling pathway, we postulated that PI3K was a molecular target of LicoA. Moreover, because LicoA marginally suppressed sUV-induced MEK1 phosphorylation, we also suspected that both MEK1 and Raf, an upstream kinase of MEK1, could be alternative molecular targets of LicoA. After examining the effect of LicoA on PI3K, MEK1, B-Raf, and C-Raf activity, we found that LicoA effectively inhibited PI3K, MEK1, and B-Raf kinase activities, but not that of C-Raf. In addition, we obtained evidence for direct physical binding of LicoA with PI3K, MEK1, and B-Raf. Moreover, LicoA bound with PI3K and B-Raf in an ATP-competitive manner, suggesting that LicoA may suppress PI3K and B-Raf activity by binding to the ATP pocket of these targets. However, LicoA appears to have an ATP non-competitive interaction with MEK1.

**Figure 5:**
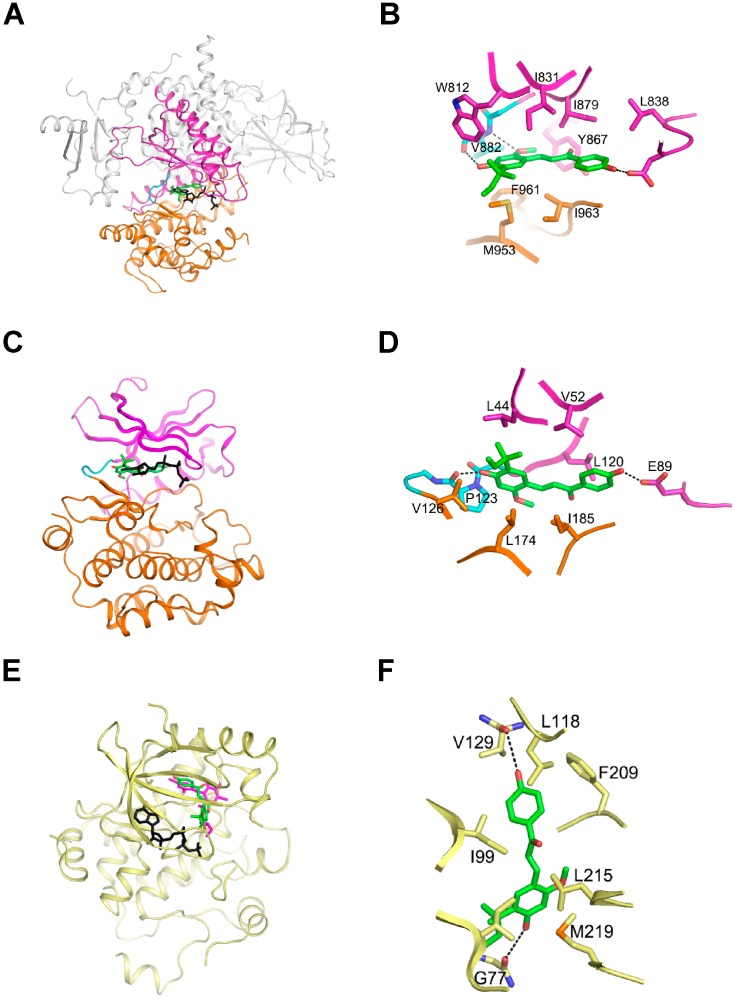
Hypothetical models of PI3K, B-RAF, and MEK1 in complex with LicoA. (**A**) and (**B**), Model structure of PI3K in complex with LicoA (**A**), and magnified view (**B**). The Ras-binding C2 domain, and the helical domain of PI3K are colored gray. LicoA (atomic structure) binds to the ATP binding site in the catalytic domain of PI3K and ATP (black) is overlaid for comparison; (**C**) and (**D**), Model structure of B-Raf in complex with LicoA (**C**) and magnified view (**D**). LicoA (atomic structure) binds to the ATP binding site of B-Raf and ATP (black) is overlaid for comparison. (**E**) and (**F**), Model structure of MEK1 (yellow) in complex with LicoA (**E**) and magnified view (**F**). LicoA (atomic structure) binds to the pocket adjacent to ATP’s (black) binding site. PD308088 (violet) has been overlaid on the model structure of MEK1-ATP-LicoA for comparison. In (**A**) and (**B**) the *N*-lobe, *C*-lobe, and hinge loop are colored violet, orange, and cyan, respectively. The residues involved in the interaction with LicoA are labeled and the hydrogen bonds are depicted as dotted lines.

Based on our observations of the ATP-competitive interaction between LicoA with PI3K and B-Raf, we initiated computer modeling studies to further explore the plausibility of this concept using the known crystal structures of PI3K and B-Raf (1, 2). PI3K consists of four domains: a Ras-binding domain, a C2 domain, a helical domain, and a catalytic domain. Although the substrate of PI3K is not a protein, the catalytic domain of the enzyme consists of an N-lobe, a C-lobe, and a hinge loop with a fold similar to protein kinases, and this structural similarity is also conserved in the ATP-binding site that is flanked by these two lobes. Consequently, ATP binds between these lobes in a manner similar to ATP binding in protein kinases. As we suspected that LicoA is an ATP-competitive inhibitor of PI3K, we performed virtual docking of the compound to the ATP binding site of PI3K (Figure 5A,B). The spatial data showed that the two hydroxyl groups of LicoA could plausibly form hydrogen bonds with the backbone carbonyl groups of Val882 in the hinge loop and Asp836 in the N-lobe of PI3K. The methoxy group could also reasonably be expected to form a hydrogen bond with the backbone amide groups of Val882. In this orientation, LicoA would be sandwiched by the hydrophobic side chains of Trp812, Ile831, Leu838, Tyr867, Ile879 from the N-lobe and Met953, Phe961, Ile963 from the C-lobe. In the modeling structure of B-Raf in complex with LicoA, the two hydroxyl groups of the compound could presumably form hydrogen bonds with the backbone carbonyl groups of Pro123 in the hinge loop and Glu89 in the N-lobe of B-Raf (Figure 5C,D). In addition, LicoA would likely form hydrophobic interactions with the side chains of Ile44, Val52, Leu120 from the N-lobe and Val126, Leu174, Ile185 from the C-lobe. A modeling study of LicoA in complex with MEK1 was carried out based on our observations that this compound inhibits MEK1 in an ATP non-competitive manner. It was predicted that LicoA may be able to interact with a pocket separated from, but adjacent to the ATP-binding site, in a manner similar to PD318088, as demonstrated in the crystal structure of the MEK1 in complex with PD318088 (3). The two hydroxyl groups of the compound would then form hydrogen bonds with the backbone carbonyl groups of Gly77 and Val127. In addition, this would allow LicoA to interact with the hydrophobic surface formed by Ile99, Leu118, Ile141, Phe209, Leu215, and Met219 (Figure 5E,F). These interactions of LicoA with MEK1 would lock MEK1 into a catalytically inactive species by stabilizing the inactive conformation, as is the case with PD318088. Further studies with X-ray crystallography to determine the actual structure of these proteins in complex with LicoA would confirm these suggestions.

**Figure 6:**
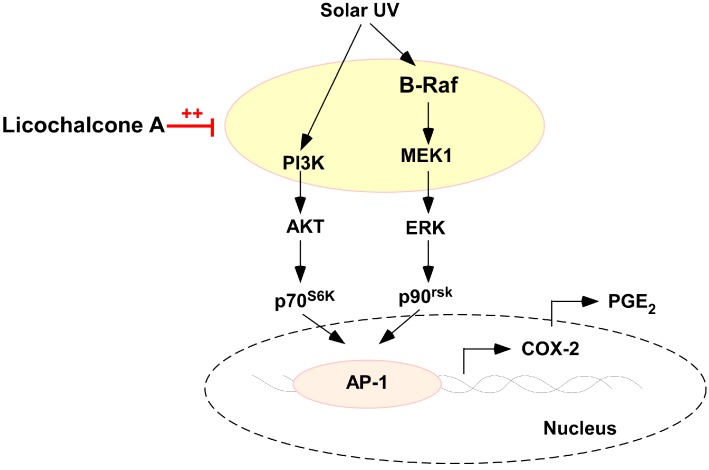
Hypothetical model for the inhibitory mechanism of LicoA against sUV-induced COX-2 expression. Red line, ++, dotted cycle

In summary, LicoA inhibits sUV-induced COX-2 expression and PGE2 generation more potently than Gc in HaCaT cells. This inhibition appears to be mediated primarily via the blockage of the Akt/mTOR and ERK1/2/p90RSK signaling pathways and subsequent suppression of AP-1 activity, rather than the inhibition of COX-2 enzyme activity itself (Figure 6). LicoA binds with PI3K, MEK1, and B-Raf and significantly inhibits their kinase activity. Collectively, these results suggest that PI3K, MEK1, and B-Raf are major molecular targets of LicoA for the suppression of sUV-mediated skin cancer. We believe these observations provide important insights into the biological actions of LicoA and the molecular basis for the development of a new chemopreventive agent.

## 3. Experimental Section

### 3.1. Materials

Licochalcone A and Glycyrrhizin were purchased from Sigma-Aldrich (St. Louis, MO, USA); Eagle’s minimum essential medium (MEM), fetal bovine serum (FBS), and l-glutamine were purchased from Gibco BRL (Carlsbad, CA, USA); and antibodies against phosphorylated Akt (Ser308), total Akt, phosphorylated mTOR, total mTOR, phosphorylated JNK1/2 (Thr183/Tyr185), total JNK, phosphorylated c-Jun (Ser73), phosphorylated p38, total p38, phosphorylated Elk, total Elk, c-Jun, phosphorylated MEK1, total MEK1, phosphorylated p90^RSK^, and total p90^RSK^ were purchased from Cell Signaling Technology (Danvers, MA, USA). Antibodies against phosphorylated ERK1/2 (Thr202/Tyr204), and total ERKs were purchased from Santa Cruz Biotechnology (Santa Cruz, CA, USA). Active PI3K, active MEK, active B-Raf, and active C-Raf were obtained from Merck Millipore (Billerica, MA, USA). Phosphatidylinositol, CNBr-Sepharose 4B, and [γ-^32^P]ATP were purchased from GE Healthcare (Piscataway, NJ, USA). The PGE_2_ assay kit was purchased from Cayman Chemicals (Ann Arbor, MI, USA). A protein assay kit was obtained from Bio-Rad Laboratories (Hercules, CA, USA). G418 and the luciferase assay substrate were purchased from Promega (Madison, WI, USA).

### 3.2. Cell Culture

HaCaT human keratinocytes (HaCaT cells) obtained from the American Type Culture Collection (Rockville, MD, USA) were cultured in monolayers in MEM containing 10% FBS and 1000 units of penicillin and 1 mg/mL of streptomycin at 37 °C under 5% CO_2_. HaCaT human keratinocytes was stably transfected with an AP-1 luciferase reporter plasmid and maintained in MEM that was supplemented with 5% FBS and 200 μg/mL of G418.

### 3.3. sUV Irradiation

A sUV irradiation system was used to stimulate cells in serum-free media. The sUV radiation source (Q-Lab Corporation, Cleveland, OH, USA) emitted at wavelengths of 295–365 nm, with a peak emission of 340 nm.

### 3.4. Cell Viability Assay

HaCaT cells were cultured overnight in 96-well plates (6000 cells/well) using 10% FBS in MEM and starved in 0.1% FBS medium for an additional 24 h. The media was then replaced with 0.1% FBS-MEM containing LicoA at the designated concentrations in a volume of 0.1 mL. Cells were incubated with the LicoA solutions for 24 h, before 20 μL of MTS reagent was added to each well. The extent of (3-(4,5-dimethylthiazol-2-yl)-5-(3-carboxymethoxyphenyl)-2-(4-sulfophenyl)-2H-tetrazolium) (MTS) reduction was spectrophotometrically measured 1 h later at 492 and 690 nm using a Multiskan MS microplate reader (Labsystems, Ramat-Gan, Israel). At least three independent experiments were performed.

### 3.5. Western Blot Analysis

Cells (1.5 × 10^6^) were cultured in 10-cm dishes for 48 h, and then starved in 0.1% FBS media for an additional 24 h to eliminate the influence of FBS on MAP kinase activation. The cells were then treated with LicoA (0–10 μM) or Gc (0–10 μM) for 1 h and irradiated with sUV for an additional 30 min. The harvested cells were disrupted and the supernatant fractions were boiled for 5 min, before protein concentration was determined using a dye-binding protein assay kit (Bio-Rad Laboratories) as described in the manufacturer’s manual. The lysates (50 μg) were then subjected to 10% SDS-PAGE and transferred to PVDF membranes (Merck Millipore, Billerica, MA, USA). After blotting, the membranes were incubated overnight with specific primary antibodies at 4 °C. The protein bands were visualized using a chemiluminescence detection kit (GE Healthcare) after hybridization with an HRP-conjugated secondary antibody.

### 3.6. Prostaglandin E2 PGE_2_ Assay

Cells were plated in 24-well plates, grown to 80% confluence in 500 μL growth medium for 48 h, and starved in 0.1% FBS–MEM for 24 h. Following treatment, culture medium was collected, centrifuged at 14,000 rpm for 5 min to remove cell debris, and frozen at −80 °C before analysis. The quantity of PGE_2_ released into the media was measured using a PGE_2_ enzyme immunoassay kit (Cayman Chemical). All experiments were performed in triplicate.

### 3.7. Luciferase Assay for activator protein 1 AP-1 Transactivation

Confluent monolayers of HaCaT cells that were stably transfected with AP-1 luciferase reporter plasmids were trypsinized, and 8 × 10^3^ viable cells suspended in 100 μL of 5% FBS/MEM were added to each well of a 96-well plate and incubated at 37 °C in a humidified atmosphere of 5% CO_2_. When the cells reached 80%–90% confluence, they were starved by culturing in 0.1% FBS MEM for an additional 24 h. The cells were then treated for 1 h with LicoA (0–10 μM) or Gc (0–10 μM) and irradiated with sUV for 6 h. After treatment, the cells were disrupted with 100 μL of lysis buffer (0.1 M potassium phosphate buffer (pH 7.8), 1% Triton X-100, 1 mM dithiothreitol (DTT), and 2 mM EDTA), and the level of luciferase activity was measured using a luminometer (Luminoskan Ascent; Thermo Electron, Helsinki, Finland).

### 3.8. PI3K Assay

Active PI3K protein (100 ng) was incubated with LicoA or LY294002 at the indicated concentrations for 10 min at 30 °C. The mixtures were then incubated with 20 μL of 0.5 mg/mL phosphatidylinositol (Avanti Polar Lipids, Alabaster, AL, USA). After 5 min at room temperature, the mixtures were incubated with reaction buffer (100 mM HEPES (pH 7.6), 50 mM MgCl_2_, and 250 μM ATP containing 10 μCi of [γ-^32^P]ATP) for an additional 10 min at 30 °C. The reaction was stopped by adding 15 μL of 4 N HCl and 130 μL of chloroform:methanol (1:1). After vortexing, 30 μL of the lower chloroform phase was spotted onto a 1% potassium oxalate-coated silica gel plate that had been previously activated for 1 h at 110 °C. The resulting ^32^P-labeled phosphatidylinositol-3-phosphate (PIP) was separated by thin layer chromatography and the radiolabeled spots were visualized by autoradiography.

### 3.9. MEK1, B-Raf, and C-Raf Kinase Assays

The *in vitro* MEK1, B-Raf, and C-Raf assays were performed in accordance with the instructions provided by Merck Millipore. Briefly, for MEK1, B-Raf, and C-Raf assays, 5 ng of active MEK1, 2 ng of B-Raf, or 5 ng of C-Raf recombinant protein and LicoA (5 and 10 μM) were incubated at 30 °C for 10 min. For each reaction, 5 μL of 5X kinase buffer [250 mM Tris/HCl (pH 7.5), 0.5 mM EGTA, 0.5% 2-mercaptoethanol], 5 μL of 500 μM ATP, and 2.25 μg of the inactive ERK or MEK1 was added. The reaction mixtures were incubated at 30 °C for 15 min. A 5 μL aliquot was removed from the reaction mixture, and added to 10 μL of 2 mg/mL of MBP substrate peptide, 5 μL of 5 × kinase buffer, and 5 μL of 0.16 μCi/μL [^32^P] ATP solution, and incubated at 30 °C for 15 min. Aliquots of 20 μL were then transferred onto p81 filter paper and washed three times with 1% phosphoric acid for 5 min per wash and once with acetone for 5 min. Radioactive incorporation was determined using a scintillation counter (LS6500; Beckman Coulter, Danvers, MA, USA). Each experiment was performed three times.

### 3.10. Immunoprecipitation Assays

The recombinant PI3K (100 ng), MEK1 (200 ng), and B-Raf (200 ng) proteins were incubated with LicoA-conjugated Sepharose 4B (or Sepharose 4B alone as a negative control) beads (100 μL, 50% slurry) in immunoprecipitation reaction buffer (50 mM Tris–HCl (pH 7.5), 5 mM EDTA, 150 mM NaCl, 1 mM dithiothreitol (DTT), 0.01% Nonidet P-40, 0.02 mM phenylmethysulfonyl fluoride) containing 2 μg/mL bovine serum albumin and 1× protease inhibitor mixture at 4 °C with gentle rocking overnight. The beads were then washed five times with immunoprecipitation reaction buffer, and the protein bound to the beads was analyzed by Western blotting.

### 3.11. ATP and LicoA Competition Assays

Briefly, 100 ng of active PI3K or 200 ng of active MEK1 or B-Raf was incubated with 100 μL of LicoA-Sepharose 4B or 100 μL of Sepharose 4B identical to the reaction buffer used in the *in vitro* immunoprecipitation assay for 12 h at 4 °C, and ATP was added at different concentrations (10 and 100 μM) to a final volume of 500 μL for 30 h. The samples were washed, before proteins were detected by Western blotting.

### 3.12. Molecular Modeling

The crystal coordinates of PI3K (PDB entry 1E8X), B-Raf (PDB entry 3C4E), and MEK1 (PDB entry 1S9J) were used for the docking of licochalcone A. Insight II (Accelrys Inc, San Diego, CA, USA) was used for the modeling study and structure analysis.

### 3.13. Statistical Analysis

Data are expressed as mean ± S.D. Student’s *t*-test was used for single statistical comparisons, with a probability of *p* < 0.05 as the criterion for statistical significance.

## Abbreviations

Gc: glycyrrhizin
LicoA: licochalcone A
sUV: solar ultraviolet
COX: cyclooxygenase
ATP: adenosine triphosphate
mTOR: mammalian target of rapamycin
PIP: phosphatidylinositol-3-phosphate
PI3K: phosphatidylinositide 3-kinase
PGE_2_: prostaglandin E2
AP-1: activator protein 1
ERK1/2: extracellular signal-regulated kinases
RSK: p90 ribosomal protein S6 kinase
MEK: mitogen-activated protein kinase kinase
JNK: c-Jun *N*-terminal kinases
MAPKs: mitogen-activated protein kinases
